# Petro. and Kali—Post-partum Derangements; Calc.—Headache; Bry.—Cough; Castoreum—Abdominal Complaints; Sulph.—Piles; Ranun. Scel.—Corns

**Published:** 1881-10

**Authors:** E. W. Berridge

**Affiliations:** London


### CLINICAL CASES.

*Petroleum and Kali*—Post-partum Derangements; *Calc.*—Headache; *Bry.*—Cough; *Castoreum*—Abdominal Complaints; *Sulph.*—Piles; *Ranun. scel.*—Corns.

E. W. Berridge, M. D., London.

*Petroleum and Kali in Post-partum Derangements.*—October 23d, 1870. Mrs. —— was confined fourteen days ago of one child. Ever since, when in bed at night in a dark room, has had a delusion that there is another baby in the bed which requires attention. She had this delusion in a former confinement, and in another a delusion that she had a third leg, which would not remain quiet. For seven days single, sharp shoots about twice a day, from upper dorsal spine into occiput.

*Diagnosis of the remedy.*—The peculiar shooting pain is found only under *Kali.* "Delusion that he is double"—the nearest approach to the mental symptoms of the case—is found under *Petr.* and *stram.* [Since this case was cured, I have added *Anac.*, *Cann. ind.*, *Mosch.*, *Sec.*, *Sil.* and *Thuya* to the list]. As *Petro.* has also "Delusion that one limb is double," I selected it in preference to *Stram.*, on account of the *anamnesis.* In this case there were thus two conflicting key-notes, *Kali* being indicated by the *latest* symptom, but *Petro.* by the mental condition. I considered the latter of the greater importance, and gave one dose of *Petroleum*, 3000 (Jenichen).

Oct. 31st. No return of the delusion; no more shooting till 27th, when it returned twice, worse than before, and seemed to fix the head for a moment. Last night, on being frightened, had a return of the pain, and at the same moment a shoot in the lumbar spine. As the mental state had been removed, and the pain increased after a temporary cessation, I now gave the corresponding remedy, *Kali*, one dose of 4000 (Jenichen).

November 15th. Once, on being frightened, has had a slight shooting from neck to occiput.

December 3d. On two consecutive days, four or five days ago, after unusual exertion (going up lofty stairs) *repeated* shooting up into head as before, for a minute, but less severe; has had it *slightly* at times before. No shooting in lumbar spine. As the pain seemed returning, I repeated the dose of *Kali* 4000.

Dec. 12th. Has a catarrh. On waking this morning the delusion returned. Last night *very* slight shooting into head. The recurrence of the mental state, combined with the great amelioration of the pain, pointed to the first remedy again, and I gave one dose of *Petr.* 3000.

March 13th, 1871. Reports that she has only had the shooting *occasionally*, and not so severely; no other symptoms.

The above was one of those rare cases where two remedies have to be given in alternation, *according to a corresponding alternation of symptoms.* This is the alternation to which HAHNEMANN refers as being sometimes necessary. In several places where he uses the term, he distinctly states this to be his meaning; hence it is evident that he must have used it in the same sense in other passages, though he did not deem it necessary to repeat his explanation on every occasion. The modern unscientific, empirical practice of *á priori* "alternation," or rotating change of medicines *without* a corresponding change of symptoms, is quite different, and is most emphatically repudiated by HAHNEMANN as unnecessary, unsafe, and we might add, a relic of the lawlessness of allopathic polypharmacy; yet some mongrels continually misquote and pervert HAHNEMANN's plainest teachings, to excuse their own shortcomings, and to blind the eyes of their victims to the falsity of their pretensions.

*Calcarea in Headache.*—March 15th, 1870. Mrs. ——, sensation (not pain) as if the brain was gradually squeezed, then relaxed, then again squeezed, and so on; she has had this for four days, commencing earlier each day, and lasting till she went to bed; to-day it came on at 10 A. M.; it makes her feel as if she would lose her senses; feeling of rush of blood to the head; feeling as if she squinted; feeling as if she would fall when walking; giddiness; the squeezing is relieved by lying down, or by pressure with the cold hand; aggravated by strong light, reading, writing, on looking up, and when the head is covered.

*Diagnosis of the remedy.*—Feeling of losing the senses (taken as the key-note because a mental symptom): *Acon., Agar., Alum., Ambr., Bov., Bry., Calc., Cann., Carb. an., Chlorum, Laur., Magn., Mag. sul., Merc., Mercurial, Mosch., Nat. m., Plat., Sepia, Sulph., Stram., Thea.*

Squeezing of brain: *Bov., Calc., Cann., Mag. s., Nat. m., Sulph.* (with many others which have not the first symptom).

Head worse from looking up: *Calc., Sulph.*

Head worse from reading or writing, and better from lying: *Calc., Natr. m.*

*Calc.* alone corresponds to all these symptoms; it has also feeling of falling with giddiness, rush of blood to the head, and relief from pressure with cool hand; the rest have not as yet been found under it. One dose of *Calc.*, 107 M (Fincke) was given about 4 P. M.

March 17th. All the symptoms were better the same day; the next day nearly gone; to-day quite gone.

June, 1870. There has been no return.

This case again conclusively proves the absolute necessity of having a collective of conditions. *Calc.* has not produced all these symptoms in combination with these conditions, but it possesses them all separately; without such a collective, how then could the remedy have been selected? It also shows that too much reliance must not be placed on concomitants; they are sometimes of value in deciding the choice between similar remedies; nevertheless, a medicine will often cure a group of symptoms if it has produced them either separately or in other combinations, as pointed out long ago by Constantine Hering.

*Bryonia in Cough.*—May 22d, 1869. Harriet D——, æt. nine months. Has had a cough for three weeks, coming on after eating, drinking, anger, etc., in the room, sometimes lasting thirty minutes at a time; has been losing flesh for two weeks, and is very thin; irritable temper; no teeth yet; all her brothers and sisters cut their teeth late.

*Diagnosis of the remedy.*—Cough from anger: *Acon.*, *Ars.*, *Bry.*, *Cham.*, *Chin.*, *Ign.*, *Nux*, *Sep.*, *Staph.*, *Verat.*

Cough in the room: *Arg.*, *Bry.*, *Croc.*, *Laur.*, *Magn. c.*, *Magn. m.*, *Nat. mur.*, *Puls.*, *Spig.*

Cough from eating: *Anac.*, *Ant. t.*, *Ars*, *Bell.*, *Bry.*, *Calc.*, *Carb. v.*, *Caust.*, *Cham.*, *Chin.*, *Cocc.*, (*Dig.*), *Ferr.*, *Hyos.*, *Ipec.*, *Kali*, *Laur.*, *Magn. m.*, *Mosch.*, *Nux*, *Op.*, *Phos.*, (*Puls.*), *Rhus*, *Ruta*, *Sep.*, *Staph.*, *Sulph.*, *Zinc.*

Cough from drinking: *Acon.*, *Arn.*, *Arsen.*, *Bry.*, *Carb. v.*, *Chin.*, *Cina*, *Cocc.*, *Dros.*, *Fer.*, *Hep.*, *Hyos.*, *Laur.*, *Lyc.*, *Nat. mur.*, *Nux*, *Op.*, *Phos.*, *Rhus.*

*Bry.*, therefore, was the only remedy which corresponded to the cough symptoms, and also to the mental condition, and one dose (a globule) of 2000 (Jenichen) was given at once.

May 24th. Has slept much more; no cough on taking food; less irritable, and when she is angry, the cough is less severe than before; does not cough so often, or for more than three or four minutes at a time.

June 2d. Looks quite happy and lively; only occasional cough at night; gaining flesh.

September 10th. Has been well ever since.

*Castoreum in Abdominal Complaints.*—July 6th, 1871. Miss ——. For a week, and getting worse, pain in stomach going round to left hypochondrium and through to back, with shooting in left hypochondrium; this comes on every day after dinner, which is at 2 P. M. Pain in stomach better by bending double; pain in back worse by deep inspiration. *With* the pain, yawning, faintness, chilliness, borborygmus and tasteless eructations. This afternoon there was also, *with* the pain, dyspnœa and wheezing for a time, and after this had passed off there was nausea in stomach, with objective heat of forehead and cold hands. This case illustrates the selection of the remedy according to the *concomitants,* which must not be neglected, though too much stress must not be laid on them. In the abdominal chapter of the Cypher Repertory, I found that *Castoreum* corresponded best to the above conditions. (The symptoms given by Allen are, "Pain in abdomen relieved by warmth, pressing upon and *bending the body;*" "Violent pain in bowels, *arresting breathing, with yawning;*" "Piercing pain in abdomen, *with rolling about,* frequently intermitting, *with chilliness,* from 11 A. M. to 4 P. M.") I gave one dose of 200 (Leipzig) at 10.35 P. M. The yawning decreased at once.

June 7th. Slight pain in stomach in morning for a time; pain returned at 7 P. M., after tea, but much less, not felt in back; very little yawning; no other symptoms.

June 8th. Pain in stomach returned at 11 A. M. for one and a half hours; none in evening.

June 9th. Pain in stomach and left hypochondrium (but no shooting) for half an hour, one hour after breakfast and in afternoon; no other symptoms.

June 10th. Quite well; remained so.

*Comments.*—This case illustrates well the action of *Castoreum,* a drug which has been denounced as worthless and inert. I have found *yawning in connection with diarrhœa and other abdominal complaints to be characteristic thereof.*

*Sulphur in Piles.*—November 23d, 1871. Mrs. ——, æt. 30. Subject to piles for four or five years; two years ago they were bad for three days. Was confined seven weeks ago, and piles have been worse since; very bad for last month. They are now internal, but at first were external. During and after stool, throbbing, burning and smarting in piles, with shooting upwards which catches the breath; also dull aching in coccyx and sacrum, extending round sides of pelvis; all this lasts for six or seven hours after stool. Stool once in from two to four days, otherwise natural. The pain makes her feel faint, trembling, sick, inclined to move about, hot, and as if she would lose her senses; the pain at anus is better when standing than when lying. Has taken, by the advice of a *professed* homœopathic physician, *Acon.*, *Nux* 3 and *Sulph.* 3, without relief.

*Diagnosis of the remedy.*—Bœnninghausen's Repertory gives "shooting in rectum stopping the breath, *Sulph.*" This I took as the key-note or starting point, and finding that this remedy corresponded fairly with the other symptoms, I gave one dose of CM (Fincke).

Nov. 29th. Piles gone; pain much less, lasting only two hours after stool; still costive; pain was bad yesterday, but better all the other days; much less weakness, trembling, heat, and feeling of losing senses; the shooting does not catch the breath so much.

April 10th, 1872. Perfectly recovered within a week, and has remained so to this day.

*Comments.*—(1) The value of key-notes or characteristics is here shown; a key-note is not a symptom on which we prescribe *without* reference to the rest, but it is a symptom so characteristic of the remedy, that we almost always find the remaining symptoms covered by it also; hence a knowledge of key-notes saves much trouble in the selection of a remedy. (2) The superiority of the high over the low potencies is also shown. It also proves that Fincke's fluxion potencies are *not* low potencies, as some have ignorantly or maliciously asserted; else how would they cure after the *bona fide* low potencies failed?

*Ranunculus sceleratus in Corns.*—March 25th, 1872. For four months or more I had two corns on the balls of first and second left toes, sensitive to touch or pressure, with smarting and burning, and occasionally shooting; very painful on letting leg hang down, when they also throb, and especially painful by flexing toes; better by extending toes, and by wearing a *thick-soled* boot; at times numbness in the corns; knocking toes against anything so as to cause the boot to grate against the corns causes great pain and burning; the corns make me limp and hinder walking, running is out of the question. Cutting the corns and wearing plasters had only relieved temporarily; *Ant. crud.* did nothing; *Puls.* and *Baryt.* relieved for a time only; this morning they were worse than ever, I could hardly walk, the pain was at times so violent as almost to make me cry out, and the burning was like fire.

*Diagnosis of the remedy.*—Bœnninghausen's Repertory gives the following:

Corns on balls of toes: *Ant. cr., Ranunc. scel.*

Corns, burning: *Ranunc. scel.* (and six others).

Corns, shooting: *Ranunc. scel.* (and two others).

I took one dose of *Ranunc. sceleratus*, 200 (Leipzig.) In the evening was so much better that I took a long walk without inconvenience.

Nov. 26th. Pain nearly gone; could walk, and in the evening, being rather late at an appointment, suddenly found, to my astonishment, that I was running. After this, they soon got well. The corns remained for some time, but gave me no inconvenience. Afterwards they also disappeared, and I have never since been troubled with them to this day, May, 1881.

